# Integrated Eco-Health approach significantly reduces helminth infections in endemic Khong islands with emphasis on *Schistosoma mekongi*

**DOI:** 10.1186/s40249-024-01226-z

**Published:** 2024-08-02

**Authors:** Somphou Sayasone, Youthanavanh Vonghachack, Shang Xia, Shan Lv, Xiao-Nong Zhou, Peter Odermatt

**Affiliations:** 1 Lao Tropical and Public Health Institute, Ministry of Health, Vientiane Capital, Lao PDR; 2 Faculty of Basic Sciences, University of Health Sciences, Vientiane Capital, Lao PDR; 3 National Institute of Parasitic Diseases at China CDC (Chinese Center for Tropical Diseases Research), NHC Key Laboratory of Parasite and Vector Biology, WHO Collaborating Centre for Tropical Diseases, Shanghai, 200025 PR China; 4 Department of Public Health and Epidemiology, Swiss Tropical and Public Health Institute, Allschwil, Switzerland; 5 University of Basel, Basel, Switzerland

**Keywords:** *Schistosoma mekongi*, *Opisthorchis viverrini*, Soil-transmitted helminth, Parasite control, Water, Sanitation and hygiene, Eco-Health/One-Health, Lao PDR

## Abstract

**Background:**

Helminth infections, including *Opisthorchis viverrini*, hookworm, and *Trichuris trichiura*, are prevalent in Khong district, Champasack province, southern Lao People’s Democratic Republic (PDR). Schistosomiasis caused by *Schistosoma mekongi* is of public health concern on the islands of the Khong district. This study aimed to assess the impact of an Eco-Health/One-Health approach in combination with mass drug administration (MDA) to reduce these helminth infections.

**Methods:**

We conducted a community intervention using a stepped-wedge trial approach on two endemic islands (Donsom and Donkhone) of the Khong district, Champasack province, Lao PDR, between April 2012 and March 2013. In each study village, 30–40 households were randomly selected. All members of selected households, who were at home during the study period were invited to participate in the study. A baseline study was conducted to assess helminth infections, knowledge attitudes and practices toward *Schistosoma mekongi* infection, behavior of open defecation and availability of latrine at home. After the baseline (T0), the Eco-Health/One-Health approach was implemented on Donsom (intervention) and Donkhone island (control). An assessment was conducted in 2014 (T1), one year after the completion of intervention implementation, to assess the short-term impact of the Eco-Health/One-Health approach on helminth infections and compare intervention and control islands. Later in 2015, the Eco-Health/One-Health approach was implemented on control island (Donkhone). After the implementation of intervention, the parasitological assessments were conducted annually in humans in 2015 (T2), in 2016 (T3) and in 2017 (T4), and in dogs in 2017 (T4) to evaluate the long-term impact of the intervention on helminth infections. Frequency was used to describe the prevalence of helminth infections. Logistic regression was applied to associate the KAP (knowledge, attitudes, and practices and open defecation behavior) and the reduction of helminth infections between intervention and control islands. The reduction in prevalence pre- and post-intervention was associated using a McNemar test. A two-independent sample *t*-test was applied to compare the mean eggs per gram (EPG) of helminth infections between control and intervention islands. A paired t-test test was used to compare the mean EPG of stool samples before (baseline) and after (follow-up) interventions for the two islands separately. A *P*-value lower than 0.05 was considered statistically significant.

**Results:**

Eco-Health/One-Health approach appears to be associated with reduction in prevalence of *S. mekongi* by 9.0% [odds ratio (*OR*) = 0.49, *P* = 0.003] compared to the use of mass drug administration alone (control island). Additionally, this intervention package significantly reduced *O. viverrini* infection by 20.3% (*OR* = 1.92, *P* < 0.001) and hookworm by 17.9% (*OR* = 0.71, *P* = 0.045), respectively. Annual parasitological assessments between 2012 and 2017 showed that the Eco-Health/One-Health approach, coupled with MDA, steadily reduced the prevalence of *S. mekongi* on the intervention island from 29.1% to 1.8% and on the control island from 28.4% to 3.1%, respectively.

**Conclusions:**

The study findings suggest that the Eco-Health/One-Health approach appears to be associated with a significant reduction in prevalence of *S. mekongi* and helminth co-infections, particularly hookworm and *T. trichiura*. Therefore, implementing the Eco-Health/One-Health approach in schistosomiasis-endemic areas could accelerate the achievement of national goals for transmission interruption by 2025 and elimination by 2030.

## Background

Helminthiases continue to pose a significant public health concern in the Lao People’s Democratic Republic (Lao PDR), especially in its remote regions where healthcare access is limited and environmental factors favor the persistence of these parasitic infections [1]. On the islands of the Khong district, Champasack province, southern part of Lao PDR, where we conducted the community intervention, is an endemic area for *Schistosoma mekongi* [2–4]. This *S. mekongi* infection was initially discovered in 1957 in a Laotian patient admitted to a hospital in Paris, France [5]. Later, in the early 1980s, the parasite was identified as a public health concern in the area of the lower Mekong River basin both in Lao PDR (two districts) and Cambodia [2, 6, 7]. At the time, chronic and severe cases of schistosomiasis with hepatosplenomegaly were observed at the local health facilities and in the endemic community underlining the public health impact of the infection [8–10]. The first community-based intervention employing a mass drug administration (MDA) campaign of a single oral dose of praziquantel (40 mg/kg body weight) was launched in 1989, spanning all endemic communities in the Khong and Mounlapamok districts. This yearly MDA campaigns were conducted over a decade and were interrupted in 1998 when the prevalence of schistosomiasis in most endemic communities was below 5% [2, 11]. A few years after the suspension, *S. mekongi* re-emerged in most endemic communities, necessitating the resumption of the MDA campaigns in 2007 [2, 4].

Other helminths are also highly prevalent. The food-borne trematode, *Opisthorchis viverrini,* is one such significant helminth. It is a type-1 carcinogenic helminth causing bile duct cancer (cholangiocarcinoma) in persons with chronic and heavy infections [12, 13]. Soil-transmitted helminthiases, such as hookworm, are also highly prevalent in the community in these districts, which further complicated the MDA programme currently implemented in this *S. mekongi* endemic area [3, 4].

In 2016, the Lao Ministry of Health established ambitious goals to combat schistosomiasis, aiming to reduce it to a non-public health problem by 2020, interrupt its transmission by 2025, and achieve complete elimination by 2030 [14]. However, the resurgence of *S. mekongi* in areas previously considered under control suggests that relying solely on chemotherapy-based interventions may not be sufficient to achieve these elimination goals. Our baseline study revealed that awareness about schistosomiasis among the study population was very low, and latrine coverage in these communities was also minimal. Furthermore, animals such as dogs were found to be infected with *S. mekongi*, contributing to the maintenance of the parasite’s life cycle in the environment [3]. These findings underscore the need for comprehensive control programs that integrate drug administration with other strategies, including environmental management, health education, improved sanitation, and animal treatment [15–17].

In recent years, the Eco-Health/One-Health approach, which consist of a holistic and multidisciplinary framework to addresses human, animal, and environmental health interconnectedness, have gained momentum and are widely acknowledged a highly potential interventions against complex infectious diseases, including helminthiases [18–20], transmitted in the environment. These approaches may be effective in combating *S. mekongi* and other helminths such as *O. viverrini* and hookworm, which are highly prevalent in these study settings and whose transmission dynamics are complex, involving several animals as intermediate hosts and reservoirs in the immediate human environment [21].

This study aimed to investigate the effect of an integrated control package employing the Eco-Health/One-Health approach on the helminth infections with emphasis on *S. mekongi*, in endemic islands of Khong District, Champasack province in the southern part of Lao PDR. This integrated control package might be effective in controlling these helminths and enhancing current control activities toward elimination in the close future.

## Methods

### Study design

This study was a community-based intervention study adopting a stepped-wedge cluster randomised design (SW-CRT) with islands as the cluster unit. It is well understood that the stepped-wedge cluster design is a type of crossover cluster trial where a cluster starts in the control condition and receives an intervention the later stage [22]. Given the high prevalence of *S. mekongi* identified in our baseline assessment on both islands (28.4% in the control group and 29.1% in the intervention group), the SW-CRT design is both beneficial and ethically sound since the interventions are being implemented equally across both islands.

We hypothesized that the Eco-Health/One-Health approach would significantly impact *S. mekongi* and other helminth infections of public health importance, such as *O. viverrini*, hookworm, and *Trichuris trichiura*, supplementing the conventional MDA approach. Primary study outcomes included the prevalence of *S. mekongi* infection, intensity of infection, and knowledge, attitudes, and practices related to its transmission. Secondary outcomes comprised the prevalence and intensity of *O. viverrini*, hookworm, and *T. trichiura* infections.

**Fig. 1:**
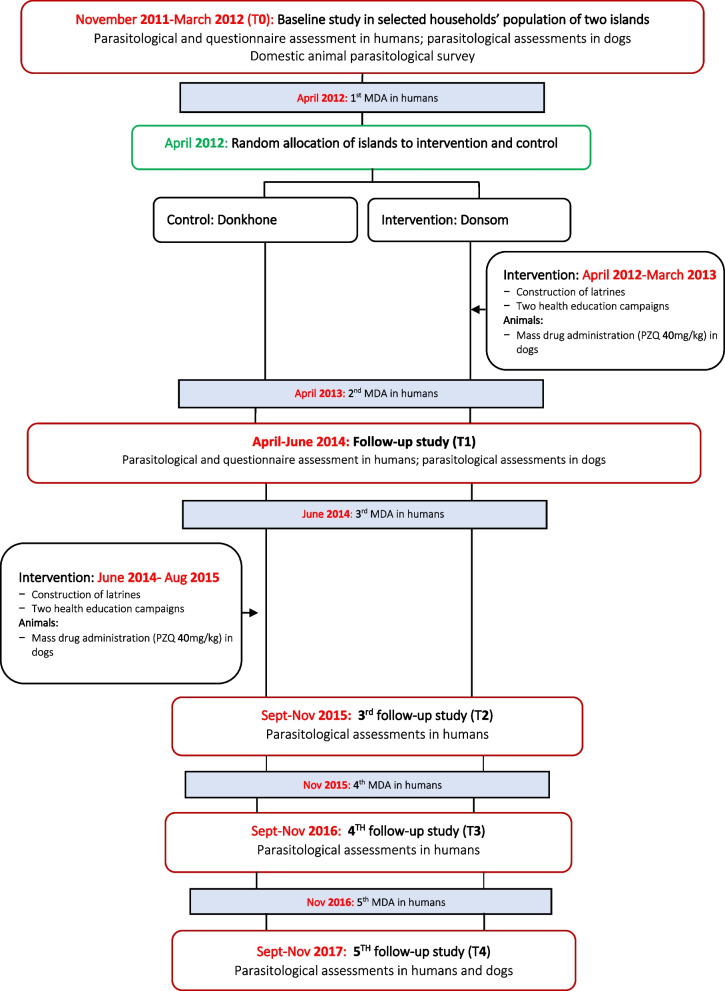
Study design

The trial was executed in two phases (Fig. 1) on the Donsom and Donkhone islands in Khong district, Champasack province, Southern Lao PDR, known as endemic islands for schistosomiasis and other helminthiasis such as *O. viverrini*, hookworm, and *T. trichiura* [2]. This phase 1 spanned from 2011 to 2014, during which the baseline (T0) was conducted to collect socio-economic data, knowledge, attitude, and practice towards schistosomiasis prevention and personal hygienic behaviour and the helminth infections in humans and animals on both islands. In each study village, about 30–40 households were randomly selected, and all members of these households aged 2 years and older present on the survey day were invited to participate in the study. In addition, all dogs owned by the selected households were also enrolled for parasitological assessment. Following this T0, two islands were randomly assigned to either the intervention (Donsom) or control (Donkhone) groups by the research team. The intervention group received the Eco-Health/One-Health approach in addition to the traditional MDA (one time per year), while the control group received only the MDA. One year after the completion of the intervention, the assessment study (T1) was conducted to evaluate the impact of the intervention compared to the baseline (T0). This follow-up assessment was carried out with the same individuals who were initially enrolled and had completed the baseline study.

The phase 2 of the trial was initiated in 2015, with Eco-Health/One-Health approach implemented on the control island (Donkhone), and the parasitological assessment was conducted annually in 2015 (T2), in 2016 (T3), and in 2017 (T4) in humans. The same households (30‒40 per village) that were included at the initial time point (T0) continued to participate in these parasitological assessments throughout the project. However, the follow-up shifted from an individual-based to a household-based assessment. Members of the selected households who were aged 2 years and older and were present at home during the assessment were invited to participate in the study. On each island, we invited 350 participants to achieve our target sample size of 622 individuals. While in dogs the assessment was only done at the endline in 2017 (T4).

### Study area and population

Khong district is one of ten districts in Champasack province, the biggest southern province of Lao PDR. This district has some 100,000 people and is located in the south of the province, at a distance of about 120 km from Pakse, the main city of Champasack province [23]. For this, two islands (Donsom and Donkhone) known as endemic areas for schistosomiasis in the Khong district were selected as the study setting [3]. Donsom has five villages with 378 houses and 2344 villagers, and three villages (Veunsom, Somven-tok, and Somven-ok) were enrolled in the study. Donkhone has three villages (Khone-neua, Khone-tai, and Hangkhone) with 260 houses and 1560 villagers and all three villages were enrolled.

### Sample size calculation

We estimated that the prevalence of *S. mekongi* on the control island would remain unchanged at 30%, as detected by the Kato-Katz method. In contrast, on the intervention island, we projected a prevalence of 20%, resulting in a difference in prevalence of 10% after one year of follow-up assessment. We set the precision at 5% and aimed for a statistical power of 80%. We used a confidence level of 95% (a Z-*alpha* of 1.96 and a Z-*beta* of 0.84), and applied a design effect of 1.1 to account for the cluster design. Based on these parameters, we calculated the minimum required samples per island of 296 participants. Anticipating a 10% loss to follow-up in one year’s assessment the intervention, we adjusted the required sample size to 326 participants per island, or a total of 622 participants across both islands.

### Development and implementation of intervention package

We developed our Eco-Health/One-Health approach in consultation with key stakeholders in a workshop held on April 15, 2012, at the Khong district governor’s office. Representatives from the national, provincial, district, and community levels were invited and joined this workshop. In this workshop, the findings of the baseline assessment were presented and discussed. Thereafter, potential community interventions were proposed, and their adequacy, applicability, and acceptability were discussed. The following Eco-Health/One-Health approach was retained, consisting of three components. First, a comprehensive latrine construction programme promotes and encourages people to build and use latrines. Second, educate villagers about the target diseases and their prevention through effective health education. Third, the MDA with praziquantel (40 mg/kg) for all dogs in the study villages. In addition, a MDA in humans was conducted on both intervention and control islands, targeting all populations aged 4 years or older according to national guidelines [24, 25]. The treatment consisted of a single oral dose of praziquantel (40 mg/kg body weight) and a single oral dose of albendazole (400 mg).

The intervention was carried out by local institutions and communities. The construction of latrines on the study islands was the full responsibility of each household participating in the study, with minimal material subsidies provided by the project (e.g., toilet bowls, septic tanks, and metal roofs). The construction process was closely supervised by village authorities, with technical support from the Division of Environment Management and Water Supply of the Provincial Health Department in Champasack province. MDA in humans was performed by medical staff from the Provincial Station for Malariology, Parasitology, and Entomology of the Provincial Health Department, as well as the District Health Office. In the case of dogs, MDA was conducted by animal health personnel from the Provincial Department of Agriculture and Forestry, as well as the Khong District Office of Agriculture and Forestry in Champasack province and Khong district, respectively. Lastly, health education campaigns were conducted by the research team and medical officers from the Division of Hygiene and Health Promotion at the Provincial Health Department in Champasack province.

### Questionnaire survey and parasitological procedures

We employed two questionnaire forms to collect the epidemiological data and two standard parasitological methods such as Kato-Katz (KK) [26] and formalin-ethyl acetate concentration technique (FECT) [27, 28] to assess the helminth infections throughout the study periods (phase 1 and phase 2). Detailed questionnaire surveys and parasitological examination procedures were outlined by Vonghachack and colleagues (2017) [3]. In brief, a household questionnaire introduced to a head of each household. This questionnaire was designed to gather data on various aspects. These included the characteristics of the household, such as the type of house, toilet facilities, and water supply. It also covered the ownership of assets, including items like farm engines, boats, cars, motorbikes, electricity, televisions, bicycles, telephones, and agricultural land. Additionally, it included information on the types of animals owned by the household, such as buffaloes, cows, goats, pigs, cats, and dogs. Moreover, an individual questionnaire was applied to each member of the study household. This was designed to gather demographic information such as age, gender, level of education, and professional activities. It also collected data on knowledge about schistosomiasis, its transmission route, its endemic area, and personal behavioural risks, including food consumption habits, contact with water, animal rearing practices, and open defecation and hygiene practice. For children under the age of 10, their parents or legal guardians provided the necessary responses.

From each human participant, two stool samples were provided during baseline and all follow-up studies. Each stool, two Kato-Katz smears were prepared, resulting in a total of four smears, which were then examined by experienced laboratory technicians after 15 min of preparation. For each dog, two grammes of stool sample were collected and preserved in a 10% formalin solution. The preserved stool samples were transported to Lao TPHI’s laboratory in Vientiane, where they processed and analyzed by experienced laboratory technicians using FECT. All helminth eggs detected under the light microscope were counted and recorded separately for each helminth species. Quality control measures were implemented, involving a senior laboratory technician reviewing 10% of the reading slides. Any discrepancies found in slide readings were addressed through consensus findings among laboratory technicians.

### Data management and analysis

All questionnaires and data forms were double-checked for completeness and consistency of the data by a senior researcher at the Lao TPHI before data entry. EpiData software, version 3.2 (Epi-Data Association; Odense, Denmark), was utilized for the data entry. Two data clerks were employed for a double data entry. The validation of data entry was performed to ensure accuracy and completeness. The cleaned dataset was exported to the STATA software, version 14, (Stata Corporation, College Station, United States) for statistical analysis.

The household’s socio-economic wealth index was constructed based on the household’s asset based approach, which was widely used, and details of the construction were previously described elsewhere [3, 4, 29]. The household’s socioeconomic status (SES) was classified into five wealth quintiles based on their cumulative standardised asset scores: (i) most poor, (ii) very poor, (iii) poor, (iv) less poor, and (v) richest. The age of participants was categorised into five groups: (i) ≤ 9 years, (ii) 10–17 years, (iii) 18–37 years, (iv) 38–49 years, and (v) ≥ 50 years. The impact of intervention on helminth infections associated with the intervention package was calculated using the percentage of the reduction in prevalence in the intervention group minus the percentage of the reduction in prevalence in the control group. The arithmetic mean with a 95% confidence interval was used to calculate egg counts per gramme of stool sample.

The coverage of each MDA was calculated using the number of targeted inhabitants who received the treatment divided by the total number of eligible inhabitants (aged 4 years and older) for MDA multiplied by 100 and stratified by setting. The *χ*^2^ test was performed to examine the different distribution of the baseline socio-demographic characteristics between the control and intervention groups. Logistic regression was applied to associate the KAP (knowledge, attitudes, and practices and open defecation behavior) and the reduction of helminth infections between intervention and control islands. The reduction in prevalence pre- and post-intervention was associated using a McNemar test. A two-independent sample *t*-test was applied to compare the mean eggs per gram (EPG) of helminth infections between control and intervention islands. A paired t-test test was used to compare the mean EPG of stool samples before (baseline) and after (follow-up) interventions for the two islands separately. A *P*-value lower than 0.05 was considered statistically significant.

## Results

### Phase 1 assessment

#### Baseline characteristics of participants

Of the 994 study participants who completed the baseline study, 621 participants submitted stool samples and completed all required processes for the first follow-up in 2014. Of these 621 participants, 364 and 257 were from the intervention and control islands, respectively. More male participated in the study (50.8% vs. 49.2%) on the intervention island, while females dominated on the control island (57.6% vs. 42.4%, *P* = 0.039). The age group was not statistically different of participants in intervention and control villages (*P* = 0.062). The mean number of household members was 5.4 on the intervention and 5.9 on the control island (*P* = 0.007). The percentage of households in the poorest quintile was 25.6% and 15.7% (*P* < 0.001) on the intervention and control island, respectively (Table 1).

**Table 1:** Baseline characteristics of study participants from Donsom (intervention) and Donekone (control) islands, Khong district, Champasack province

**Indicators**	**Overall**	**Intervention**	**Control**	*χ*^2^	*P-*value
	% (*n*), *n* = 621	% (*n*), *n* = 364	% (*n*), *n* = 257		
**Demographics**					
Female gender	53.0 (329)	54.7 (180)	45.3 (149)	4.28	0.036
**Age in years**					
Mean age (95% *CI*)	29.7 (28.0; 31.3)	28.7 (26.6; 30.7)	31.1 (28.4; 33.9)	1.40	0.062
Age group					
≤ 9	22.1 (137)	23.1 (84)	20.6 (53)		
10–17	17.9 (111)	17.6 (64)	18.3 (47)		
18–37	21.9 (136)	22.0 (80)	21.8 (56)		
38–49	18.5 (115)	20.1 (73)	16.3 (42)		
≥ 50	19.6 (122)	17.3 (63)	23.0 (59)	4.02	0.403
**Educational level**					
Preschoolers	10.6 (66)	10.5 (38)	10.9 (28)		
Illiteracy	10.0 (62)	7.1 (26)	14.0 (36)		
Primary school	56.8 (353)	59.3 (216)	53.3 (137)		
Secondary school or higher	22.5 (140)	23.1 (84)	21.8 (56)	8.22	0.042
**Professional activity**					
Farmer	58.8 (365)	59.3 (216)	58.0 (149)		
Students	29.5 (183)	30.0 (109)	28.8 (74)		
Preschool children	10.3 (64)	9.9 (36)	10.9 (28)		
Government staff	1.4 (9)	0.8 (3)	2.3 (6)	2.63	0.451
**Study households**					
Mean house members	5.6 (5.4; 5.8)	5.4 (5.2; 5.6)	5.9 (5.6; 6.2)	2.69	0.007
Wealth index					
Richest	18.7 (116)	13.5 (49)	26.1 (67)		
Less poor	21.7 (135)	25.8 (94)	16.0 (41)		
Poor	19.0 (118)	16.8 (61)	22.2 (57)		
Very poor	20.3 (126)	20.3 (74)	20.2 (52)		
Poorest	20.3 (126)	23.6 (86)	15.7 (40)	26.73	< 0.001

#### Changes in knowledge, attitudes and practices

One year following the intervention, an assessment of knowledge, attitudes, and practices (KAP) was conducted. Knowledge about schistosomiasis increased by 64.5% (from 10.2% to 74.7%) in the intervention group and by 41.2% (from 15.2% to 56.4%) in the control group. This increase in knowledge was significantly higher in the intervention group than in the control group (*OR* = 2.28, *P* < 0.001). Knowledge about endemic areas increased by 37.1% (from 6.3% to 43.4%) in the intervention group and by 21.4% (from 9.7% to 31.1%) in the control group (*OR* = 1.70, *P* = 0.002). Additionally, knowledge about transmission rose by 47.8% (from 1.4% to 49.2%) in the intervention island and by 34.3% (from 3.1% to 37.4%) in the control island (*OR* = 59.00, *P* < 0.001). Practices of open defecation were reduced by 44.8% (from 58.5% to 13.7%) in the intervention and by 28.4% (from 54.5% to 26.1%) in the control (*OR* = 0.45, *P* < 0.001). Furthermore, latrine coverage increased by 44.5% (from 41.2% to 85.7%) on the intervention compared to an increase of 15.6% (from 45.5% to 61.1%) on the control island, with a significant difference (*OR* = 3.82, *P* < 0.001) (see Table 2).

**Table 2:** Changes of availability and utilization of latrines and knowledge, attitudes, and practices one year after intervention toward *Schistosoma mekongi* transmission and prevention among study participants on Donesom (intervention, *n* = 364) compared to those on Donkone (control, *n* = 257) island

**Variables**	**Baseline, 2012, % (*n*)**	**Follow-up, 2014, % (*n*)**	**% of KAP increase**	**% of actual impact**		
					***OR* (95% *CI*)**	***P*-**value
**Know about schistosomiasis**						
Control	15.2 (39)	56.4 (145)	+ 41.2	Ref	1.00	
Intervention	10.2 (37)	74.7 (272)	+ 64.5	+ 23.3	2.28 (1.62‒3.21)	< 0.001
**Know that schistosomiasis is endemic in their community**						
Control	9.7 (25)	31.1 (80)	+ 21.4	Ref	1.00	
Intervention	6.3 (23)	43.4 (158)	+ 37.1	+ 15.7	1.70 (1.21‒2.37)	0.002
**Know that animals can get schistosomiasis**						
Control	7.4 (19)	17.9 (46)	+ 10.5	Ref	1.00	
Intervention	3.9 (14)	21.4 (78)	+ 17.5	+ 7.0	1.29 (0.86‒1.91)	0.215
**Know that *S. mekongi* infection through contact with water in the Mekong River**						
Control	3.1 (8)	37.4 (97)	+ 34.3	Ref	1.00	
Intervention	1.4 (5)	49.2 (179)	+ 47.8	+ 13.5	1.59 (1.15‒2.21)	0.005
**Can *S. mekongi* be prevented (yes *vs* no)?**						
Control	22.7 (58)	52.1 (134)	+ 29.4	Ref	1.00	
Intervention	34.7 (126)	60.2 (219)	+ 25.5	-3.9	1.39 (1.01‒1.91)	0.047
**Is *S. mekongi* harmful (yes *vs* no)?**						
Control	5.8 (15)	18.7 (48)	+ 12.9	Ref	1.00	
Intervention	3.9 (14)	25.3 (92)	+ 21.4	+ 8.5	1.47 (0.99‒2.18)	0.053
**Open defecation practice for your last time (yes *vs* no)?**						
Control	54.5 (140)	26.1 (67)	-28.4	Ref	1.00	
Intervention	58.5 (213)	13.7 (50)	-44.8	-16.4	0.45 (0.30‒0.68)	< 0.001
**Availability of latrine at home (yes *vs* no)**						
Control	45.5 (117)	61.1 (157)	+ 15.6	Ref	1.00	
Intervention	41.2 (150)	85.7 (312)	+ 44.5	+ 28.9	3.82 (2.59‒5.62	< 0.001
**Often taking a bath in the Mekong (yes *vs* no)**						
Control	84.8 (218)	63.8 (164)	-21.0		1.00	
Intervention	98.4 (358)	67.0 (244)	-31.4	-10.4	1.15 (0.82‒1.61)	0.405

*OR* Odds Ratio was calculated from logistic regression model, *CI* confidence interval, *KAP* knowledge, attitude, and practice

% of actual impact = % of KAP increase in intervention—% of KAP increase in control

#### Impact of intervention on helminth infections

*S. mekongi* infection declined by 19.5% (from 29.1% to 9.6%, *OR* = 1.13, *P* < 0.001) on the intervention island compared to 10.5% (from 28.4% to 17.9%, *OR* = 0.53, *P* = 0.004) on the control island. In comparison to the control, the Eco-Health/One-Health approach further reduced the prevalence of *S. mekongi* by 9.0% (*OR* = 0.52, *P* = 0.003), in addition to the effects of the MDA. The prevalence of *O. viverrini* infection decreased by 13.7% (from 82.7% to 69.0%, *OR* = 0.35, *P* < 0.001) on the intervention island. However, on the control island, it increased slightly by 0.8% (from 52.9% to 53.7%, *OR* = 1.05, *P* = 0.831). When compared to the control, the reduction in the prevalence of *O. viverrini* was significantly greater (*OR* = 1.92, *P* < 0.001). On the intervention island, the prevalence of hookworm infection decreased significantly by 23.0% (from 54.1% to 31.9%, *OR* = 0.38, *P* < 0.001). On the control island, there was a smaller decrease of 5.1% (from 44.8% to 39.7%, *OR* = 0.71, *P* = 0.139). When comparing the intervention to the control island, the reduction in the prevalence of hookworm was significantly higher (*OR* = 0.71, *P* = 0.045). *T. trichiura* infections went down by 3.9%, from 6.2% to 2.3% on the intervention island by 3.6%, from 5.5% to 1.9% on the control island (Table 3).

**Table 3:** Impact of the ecohealth intervention package on helminth infections among inhabitants on Donsom (intervention) island compared with those on Donkhone (control) island one year after completion of the intervention (*n* = 621)

Helminth species	Baseline 2012, % (*n*)	Follow-up 2014, % (*n*)	% of differences	% of actual impact	Control vs. intervention		Baseline vs. follow-up	
					***OR* (95% *CI*)**	***P*-**value	***OR* (95% *CI*)**	***P*-**value
***Schistosoma mekongi***								
Control	28.4 (73)	17.9 (46)	-10.5	Ref	1.0		0.53 (0.33‒0.83)	0.004
Intervention	29.1 (106)	9.6 (35)	-19.5	-9.0	0.49 (0.30‒0.78)	0.003	0.13 (0.06‒0.25)	< 0.001
***Opisthorchis viverrini***								
Control	52.9 (136)	53.7 (138)	+ 0.8	Ref	1.0		1.05 (0.67‒1.63)	0.831
Intervention	82.7 (301)	69.0 (251)	-13.7	-20.3	1.92 (1.37‒2.67)	< 0.001	0.38 (0.24‒0.59)	< 0.001
**Hookworm**								
Control	44.8 (115)	39.7 (102)	-5.1	Ref	1.0		0.71 (0.44‒1.14)	0.139
Intervention	54.1 (197)	31.9 (116)	-23.0	-17.9	0.71 (0.51‒0.99)	0.045	0.35 (0.24‒0.48)	< 0.001
***Trichuris trichiura***								
Control	6.2 (16)	2.3 (6)	-3.9	Ref	1.0		0.33 (0.09‒0.96)	0.025
Intervention	5.5 (20)	1.9 (7)	-3.6	-0.3	0.82 (0.27‒2.47)	0.725	0.24 (0.06‒0.72)	0.005

*OR* Odds ratio, *CI* Confidence interval

The intensity of *S. mekongi* infection, measured by mean EPG, was significantly reduced on the intervention island by 10.5 EPG (from 18.6 EPG at baseline to 8.1 EPG at follow-up, *P* = 0.010). In contrast, the reduction in EPG intensity on the control island was two-fold lower, at 5.8% (from 11.0 EPG to 5.8 EPG), though this reduction was not statistically significant (*P* = 0.374). Notably, other helminth infections such as *O. viverrini*, hookworm, and *T. trichiura* did not exhibit a statistically significant decrease in EPG compared to baseline intensity (*P* > 0.05) (Table 4).

**Table 4:** Intensity of infections for helminth infections among inhabitants on Donsom (intervention, *n* = 364) compared with Donkhone (control, *n* = 257) island, one year after completion of the intervention

	**Baseline study, 2012**^a^			**Follow-up study, 2014**^a^			**Differences**^b^	
	Mean (95% *CI*)	*t*	***P****-*value	Mean (95% *CI*)	*t*	*P-*value	*t*	***P****-*value
***Schistosoma mekongi***								
Control	11.0 (6.1‒15.9)	1.00		5.8 (3.1‒8.5)	1.00		1.87	0.062
Intervention	18.9(11.6‒26.3)	-1.76	0.078	8.1 (3.7‒10.0)	-0.89	0.374	2.57	0.010
***Opisthorchis viverrini***								
Control	188.4 (105.1‒271.7)	1.00		140.5 (80.3‒200.6)	1.00		1.14	0.254
Intervention	1208.7 (1231.4‒2012.6)	-7.06	< 0.001	1067.8 (981.7‒1753.8)	-6.17	< 0.001	1.03	0.303
**Hookworm**								
Control	50.7 (35.9‒65.5)	1.00		66.5 (51.2‒83.9)	1.00		1.37	0.208
Intervention	232.0 (138.6‒325.3)	-3.77	< 0.001	190.4 (147.0‒233.7)	-4.37	< 0.001	1.03	0.306
***Trichuris trichiura***								
Control	0.5 (< 0.1‒1.0)	1.00		0.6 (< 0.1‒1.2)	1.00		-1.00	0.318
Intervention	0.5 (< 0.1‒1.1)	0.01	0.995	0.5 (< 0.1‒1.2)	0.18	0.858	-1.00	0.318

*EPG* Egg counts per gram stool, *CI* Confidence interval

^a^Comparison of the mean EPG of helminth infections at baseline and follow-up between control and intervention islands using a two-independent sample *t*-test

^b^Comparison of the difference in mean EPG for helminth infections between baseline and follow-up studies using a paired *t*-test

### Phase 2 assessment

#### Impact on helminth infections

**Fig. 2:**
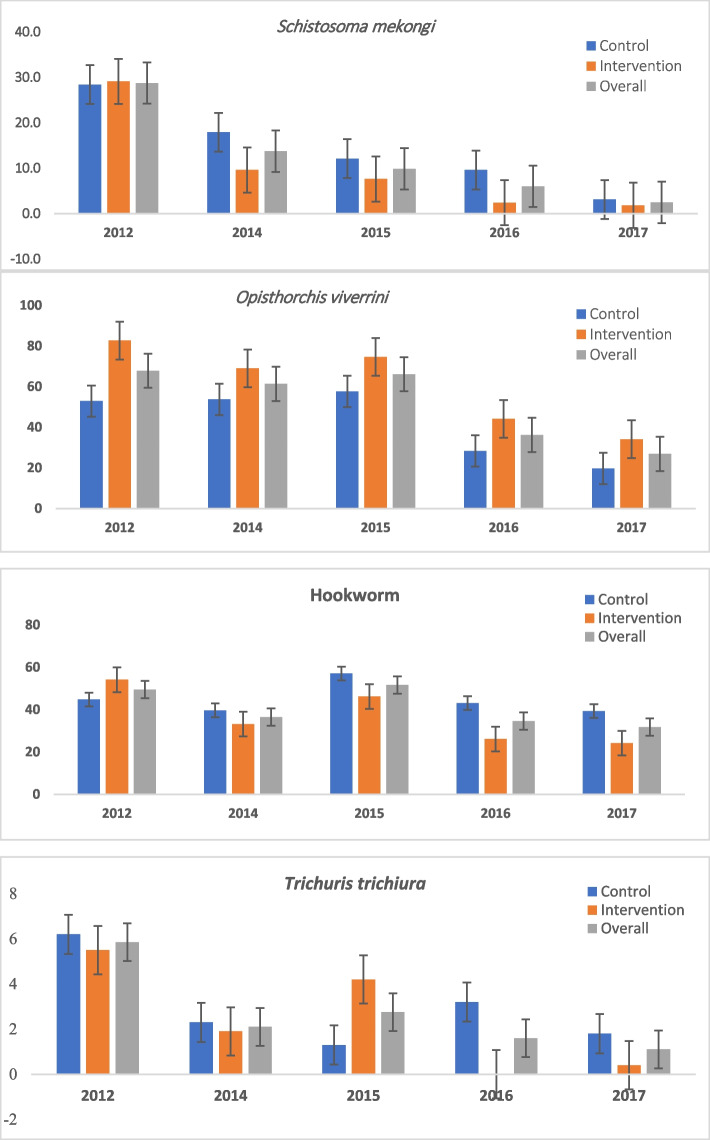
Prevalence estimates of the infection over time. Error bars show binomial confidence intervals for each estimate. The study population changed pre and post 2015. Initially, it included same individuals from study households. Post-2015, it expanded to include other household members aged 2 + . This shift may influence infection trends

Between 2012 and 2017, annual parasitological assessments consistently revealed a decline in *S. mekongi* infection among inhabitants of both islands, dropping from 28.8% in 2012 to a very low prevalence of 2.5% in 2017, marking a reduction of 26.3% (*P* < 0.001). Specifically, on the intervention island, the prevalence decreased from 29.1% in 2012 to 1.8% in 2017, while on the control island, it decreased from 28.4% in 2012 to 3.1% in 2017. Additionally, *O. viverrini* infection decreased from 82.7% to 34.1% on the intervention island and from 52.9% to 19.7% on the control island between 2012 and 2017. Hookworm infection decreased from 54.1% to 24.2% on the intervention island and from 44.8% to 39.9% on the control island during the same period. Moreover, *T. trichiura* infection decreased from 5.5% to 0.4% on the intervention island and from 6.2% to 1.8% on the control island between 2012 and 2017 (Fig. 2).

#### Latrine and MDA coverage

**Fig. 3:**
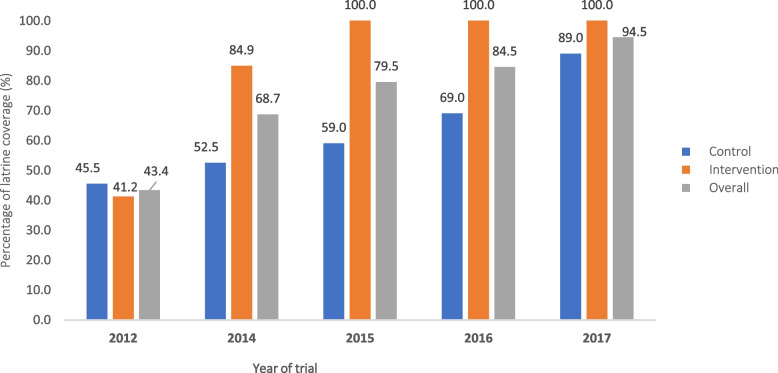
Latrine coverage on two study islands between 2012–2017: Donsom (intervention, orange) compared with Donkhone (control, blue) island, and overall (grey)

**Fig. 4:**
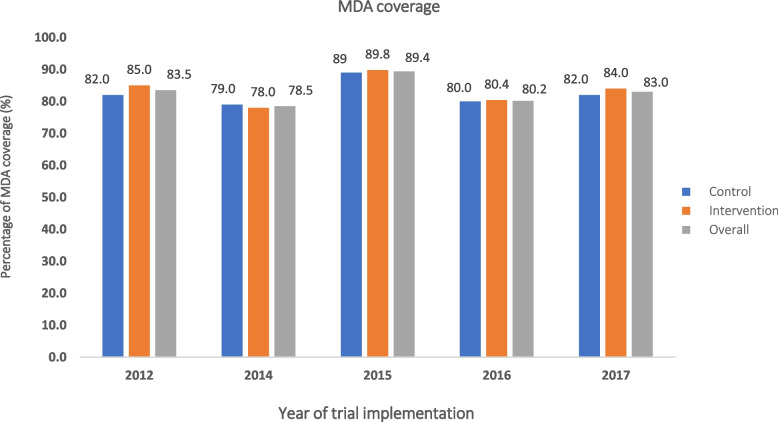
Coverage of mass drug administration on two study islands between 2012–2017: Donsom (intervention, orange) compared with Donkhone (control, blue) island, and overall (grey)

A total of 351 latrines were constructed across study villages on the two islands over the course of the project implementation (2012–2017). Latrine coverage experienced a significant increase from 41.2% in 2012 to 100.0% in 2017 (a rise of 58.8%) on the intervention island. Similarly, on the control island, the coverage increased from 45.5% in 2012 to 89.0% in 2017 (Fig. 3). Furthermore, MDA coverage remained consistently above 75.0% in both islands from 2012 to 2017 (Fig. 4).

### *S. mekongi* infection in dogs

**Fig. 5:**
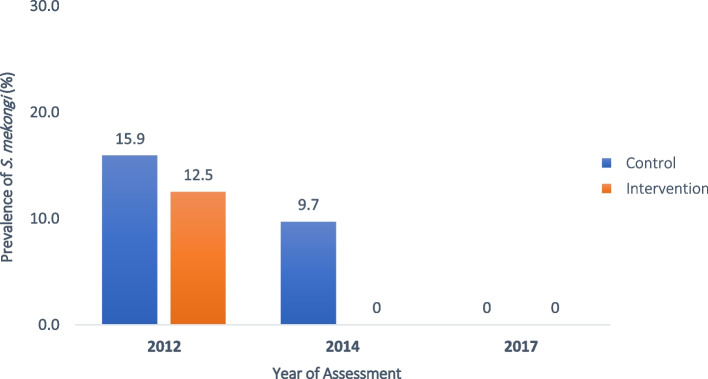
Prevalence of *Schistosoma mekongi* infection in dogs 2012‒2017 detected by stool sample analysis using formalin-ethyl acetate concentration technique

For dogs, stool samples were initially collected from 68 dogs (intervention: 24 and control: 44 dogs) at baseline in 2012. From these dogs, 12.5% (3/24) of them in the intervention and 15.9% (7/44) in the control island were infected with *S. mekongi.* During the follow-up in 2014, 70.6% (48 dogs) of these were located. Among the located dogs, 17 were from the intervention island and 31 from the control island. Analysis of stool samples revealed that all dogs on the intervention island were cleared from *S. mekongi*, while 9.7% (3/31) of dogs on the control island still tested positive. Notably, during the follow-up in 2017, no eggs of *S. mekongi* infection were detected in the study villages on both islands (Fig. 5).

## Discussion

*S. mekongi* remains a public health concern in the communities on islands in the Khong district, Champasack province, in the southern part of Lao PDR [2]. Other helminth parasites such as *O. viverrini,* hookworm and *T. trichiura* were also highly endemic. We employed a stepped-wedge cluster randomised design to assess the impact of an Eco-Health/One-Health approach including promotion of latrine construction and its use, an MDA in dogs using praziquantel and two health education campaigns in addition to annual MDA in humans in six villages on two islands (three villages each) in Khong district to control these helminth infections. A year after the intervention, a significant improvement was observed in the knowledge, attitudes, and practices (KAP) related to schistosomiasis. The intervention group showed a 64.5% increase in knowledge about schistosomiasis, significantly higher than the 41.2% increase in the control group. Knowledge about endemic areas and transmission also saw a significant rise in the intervention group, with increases of 37.1% and 47.8% respectively. The intervention package significantly reduced the prevalence of *S. mekongi* by 9.0% (*OR* = 0.49, *P* = 0.003) compared to the use of mass drug administration alone (control island). Additionally, the intervention package significantly reduced *O. viverrini* infection by 20.3% (*OR* = 1.92, *P* < 0.001) and hookworm infection by 17.9% (*OR* = 0.71, *P* = 0.045). Our trial revealed a successful installation of latrines with a coverage of over 85% in less than one year, attaining almost 100% coverage on less than one and a half years in the intervention island. Access to adequate sanitation is an essential indicator embodied in the sustainable development goals (SDG 6) on clean water and sanitation, which have been endorsed by the government of Lao PDR [30]. In recent years, the Lao Ministry of Health has made substantial efforts to achieve open defecation-free (ODF) status for all households across the country by implementing the community-led total sanitation programme [31]. However, latrine coverage varies considerably by setting, with the lowest coverage observed in the remote areas, coinciding with places wherein most households in the community faced economic disadvantage and were unable to cover the costs of the construction [31, 32]. In this study, we offered minimal subsidies to the households on the study islands, such as toilet bowls, septic tanks, and metal roofs, which considerably encouraged the contribution and participation of endemic communities in latrine construction. This could be a possible solution for a rapid uptake of latrine construction, which might increase latrine coverage to fulfil ODF status and SDG targets.

Still, the dramatic increase in latrine coverage to almost all households did not result in complete open defecation-free practices among study participants. Our follow-up showed that one in every six participants from the intervention island continued open defecation practices. The professional activities such as farming on rice fields might put them in places where no latrines are available. Nonetheless, open defecation decreased by three times when compared to the baseline observations and was significantly lower than on control island (*OR* = 0.45, *P* < 0.001). The incomplete reduction of open defecation was similar to our previous study, in which we observed that 19.5% of study participants still practiced open defecation after having a latrine in their households [33].

Our findings suggest that a thorough health education campaign is effective to increase awareness of diseases among inhabitants in the endemic communities. The health education campaigns on the intervention increased knowledge about schistosomiasis more than 23 times (*P* < 0.001) when compared to the control island. Participants who knew that schistosomiasis was endemic in their community increased by 15.7 times compared to the control island (*P* < 0.002). Participants who perceived that schistosomiasis can be prevented increased more than three times compared to the control island (*P* = 0.047). Our findings are aligned with previous studies conducted in different regions, which concluded that health education improved the participants’ KAP towards disease prevention and control, including helminthiases [17, 34]. Interestingly, it appears that the KAP among the study participants on the control island also improved. This could potentially be attributed to the annual visits by the team from the national control programme. During these visits, they likely disseminated information about schistosomiasis to the inhabitants of these endemic islands. This dissemination of information could have inadvertently influenced the control island, leading to what is known as ‘information contamination’. This highlights the complexities of conducting public health research in real-world settings and the importance of considering such factors when interpreting the results.

We found that the Eco-Health/One-Health intervention package employed in the study island in the first stage of the trial significantly reduced the *S.* *mekongi* infection in the first assessment and long-term evaluations both on prevalence and intensity of infection. These findings confirm the previous reports suggesting that an integrated intervention could be an ideal approach for driving *S. mekongi* control programmes to reach elimination goals [33, 35]. In our study, the assessment one year after the completion of the intervention implementation showed a reduction of twofold in *S. mekongi* infection in the intervention when compared to the control villages (*OR* = 0.49, *P* < 0.001). After implementation of the intervention package on the control island, the reduction in prevalence of *S. mekongi* continued steadily on both islands reaching the lowest prevalence of 1.8% for intervention and 3.1% for control in 2017, respectively.

Our study also showed a significant impact of this integrated package on *O. viverrini* and hookworm infections, which are helminths infection of public health concern in the study area and Lao PDR. Our one year of assessment revealed that *O. viverrini* infection decreased by 20.3 times in the intervention island when compared to the control island (*P* < 0.001). For hookworm, the odds of infection decreased by 17.9 times (*P* < 0.001) compared to the control island. For longer-term assessment, the reduction in prevalence was not achieved at a level of much less than 20% for *O. viverrini* and for hookworm, which is the cut-off point WHO recommended in the national strategy for selective case treatment (no MDA is required) in the endemic community [24]. The high prevalence of *O. viverrini* (i.e., intervention: 82.7% and control: 52.9%) and hookworm (i.e., intervention: 54.1% and control: 44.8%) possibly created favourable conditions for dynamic transmission, especially when coupled with continued risk practices such as raw fish consumption and agricultural cultivations of inhabitants, which resulted in heavy re-infections among study participants.

The praziquantel treatment in dogs cleared *S. mekongi* infection in the study villages on intervention island, while *O. viverrini* infection was also significantly reduced among the dogs in the invention (5.1%) and control (7.3%) villages in one year of the follow-up assessment. Indeed, while dogs are not the primary reservoir for *O. viverrini*, other animals, particularly cats, play a significant role in maintaining the lifecycle of this parasite. Studies have shown that a substantial proportion of cats in endemic areas are infected with *O. viverrini* [3]. Furthermore, dogs have been identified as a reservoir for the hookworm (*Ancylostoma ceylanicum*), which is notably prevalent in Cambodia, where more than half of the human population in certain areas harbour this parasite [36]. The unaltered infections in these animals, they continue shedding the parasite eggs into the environment in endemic settings.

The current study has several limitations. It is important to note that our study used the Kato-Katz technique to diagnose helminth infections, which did not allow us to distinguish between *O. viverrini* and minute intestinal fluke infections since the egg morphology is extremely similar. Therefore, some of the eggs reported as *O. viverrini* might be eggs of minute intestinal flukes. Because some participants were lost to follow-up in 2015, the follow-up changed from individuals to communities. This led to a rise in the number of people infected with helminths like *O. viverrini* and hookworm, since these people may not have gotten treatment during the MDA campaigns.

## Conclusions

The intervention package utilizing an Eco-Health/One-Health approach appears to be associated with a significant reduction in *S. mekongi* infection as well as other intestinal helminth infections in a phase 1 assessment on the intervention island. In the phase 2 assessment, this intervention package shows a significant effect on controlling *S. mekongi* infection and could bring the *S. mekongi* to the pre-elimination stage. Therefore, adopting this intervention package for the schistosomiasis control programme might accelerate the achievement of the national goal of transmission interruption by 2025 and elimination by 2030.

## Data Availability

All study datasets analysed for this manuscript are available from the corresponding author upon reasonable request.

## Abbreviations

Lao PDR: Lao People’s Democratic Republic
Lao TPHI: Lao Tropical and Public Health Institute
*OR*: Odds ratio
EPG: Eggs per gram
MDA: Mass drug administration
FECT: Formalin-ethyl acetate concentration technique
WASH: Water, sanitation and hygiene
